# Development and validation of nodal staging score in pN0 patients with esophageal squamous cell carcinoma: A population study from the SEER database and a single‐institution cohort

**DOI:** 10.1111/1759-7714.14670

**Published:** 2022-10-11

**Authors:** Haitong Wang, Yueyang Yang, Kai zhu, Ningning Zhu, Lei Gong, Hongdian Zhang, Mingquan Ma, Peng Ren, Yufeng Qiao, Xiangming Liu, Peng Tang, Zhentao Yu

**Affiliations:** ^1^ Department of Esophageal Cancer, Tianjin Medical University Cancer Institute and Hospital, National Clinical Research Center for Cancer, Key Laboratory of Cancer Prevention and Therapy Tianjin's Clinical Research Center for Cancer Tianjin China; ^2^ National Cancer Center/National Clinical Research Center for Cancer/Cancer Hospital & Shenzhen Hospital Chinese Academy of Medical Sciences and Peking Union Medical College Shenzhen China

**Keywords:** beta‐binomial model, esophageal squamous cell carcinoma, lymph node metastasis, lymph nodes examined, nodal staging score

## Abstract

**Background:**

Patients with esophageal squamous cell carcinoma (ESCC) with lymph node metastasis may be misclassified as pN0 due to an insufficient number of lymph nodes examined (LNE). The purpose of this study was to confirm that patients with ESCC are indeed pN0 and to propose an adequate number for the correct nodal stage using the nodal staging score (NSS) developed by the beta‐binomial model.

**Methods:**

A total of 1249 patients from the Surveillance, Epidemiology, and End Results (SEER) database between 2000 and 2017, and 1404 patients diagnosed with ESCC in our database between 2005 and 2018 were included. The NSS was developed to assess the probability of pN0 status based on both databases. The effectiveness of NSS was verified using survival analysis, including Kaplan–Meier curves and Cox models.

**Results:**

Many patients were misclassified as pN0 based on our algorithm due to insufficient LNE. As the number of LNE increased, false‐negative findings dropped; accordingly, the NSS increased. In addition, NSS was an independent prognostic indicator for pN0 in patients with ESCC in the SEER database (hazard ratio [HR] 0.182, 95% confidence interval [CI] 0.046–0.730, *p* = 0.016) and our database (HR 0.215, 95% CI 0.055–0.842, *p* = 0.027). A certain number of nodes must be examined to achieve 90% of the NSS.

**Conclusions:**

NSS could determine the probability of true pN0 status for patients, and it was sufficient in predicting survival and obtaining adequate numbers for lymphadenectomy.

## Graphical abstract

Probability density for some beta distributions.

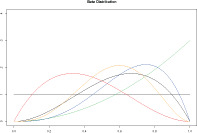

## INTRODUCTION

Esophageal cancer (EC) is the eighth most common type of cancer worldwide and the sixth primary cause of tumor‐related deaths. It is characterized by a high mortality rate and poor prognosis, with 604 100 new cases and 544 076 deaths in 2020, as reported by GLOBOCAN.^1^ Surgeries of diverse approaches are the major therapies for EC, including digestive tract reconstruction and lymphadenectomy. Esophageal squamous cell carcinoma (ESCC) is highly prevalent in Asia, particularly in China. Lymph node metastasis (LNM) is the most important prognostic factor correlated with long‐term survival in patients with ESCC^2^ and has been investigated by many studies, either on the number of harvested lymph nodes or extension of lymphadenectomy.^3^, ^4^ The recommended number of lymph nodes examined (LNE) by the American Joint Committee on Cancer (AJCC) Cancer Staging Manual 8th edition is 10 for pT1 cancers, 20 for pT2, and ≤30 for pT3/4. This is the current clinical guideline for lymphadenectomy, which was based on a study by Rizk et al., who investigated the relationship between survival and lymphadenectomy using random forest multivariate regression analysis.^5^ The recommended LNE number varied from 14 to 30, which was based on the relationship between acquired LNE and long‐term survival.^6^, ^7^ However, few studies have focused on the appropriate LNE numbers in patients who were in the pN0 stage.

Gönen et al.^8^ proposed an excellent tool based on a beta‐binomial model, called the nodal staging score (NSS), to estimate the probability of correct staging in various cancer patients at the pN0 stage. NSS has been applied in many studies and has succeeded in prostatic, endometrial, upper tract urothelial, and gastric cancers,^9^, ^10^, ^11^, ^12^, ^13^ but its significance in ESCC is ambiguous. In this study, we calculated NSS based on the beta‐binomial model, validated its effectiveness with survival information, and quantified the lymph nodes to be examined during esophagectomy using NSS.

This study aimed to establish a prediction model for accurately evaluating lymph node numbers at different T stages in patients with ESCC.

## METHODS

### Patients

The Surveillance, Epidemiology, and End Results (SEER) program is an authoritative platform of the National Cancer Institute that provides information on cancer incidence and survival from cancer registries, capturing approximately 28% of the US population. In this study, we extracted 69 083 patients with EC diagnosed between 2000 and 2017 from the latest version of the SEER database, covering 18 registries, released in November 2019, using SEER*Stat software (seer.cancer.gov/seerstat) Version 8.3.9.1. We included patients who underwent esophagectomy with a histopathological diagnosis of ESCC and excluded those with other simultaneous malignant tumors of distant metastasis diagnoses, and those uninformed about the characteristics of interest. We excluded patients with follow‐up or survival time less than 1 month. A flowchart is shown in Supporting Information Figure S1.

Another independent single‐institution Chinese database was established by Tianjin Medical University Cancer Institute and Hospital. Using the same inclusion and exclusion criteria, we included 1404 patients who underwent either Ivor–Lewis or McKeown esophagectomy with lymphadenectomy for ESCC between January 2005 and January 2018.

Clinical characteristics, including age, sex, race (not included in our database), marital status (not included in our database), differentiation grade, tumor size and site, histopathology, T stage, number of positive and examined lymph nodes, chemotherapy, radiotherapy, neoadjuvant chemotherapy, and neoadjuvant radiotherapy were extracted from the two databases. The disease‐specific survival (DSS) time was included for survival analysis. The DSS time began at the time of diagnosis and ended at the time of death caused by ESCC. Patients who did not die of ESCC were treated as loss to follow‐up.

This retrospective study was approved by the ethics committee of Tianjin Medical University Cancer Institute and Hospital in accordance with the ethical standards prescribed by the Helsinki Declaration.

### Model development

As previously described, NSS methodology has already been used for other types of cancer.^8^, ^9^, ^10^, ^11^, ^12^, ^13^ In our study, we tried to use the NSS to verify the presence of some positive lymph nodes in patients who had already been diagnosed with pN0 stage cancer. Probability can be computed using the following assumptions:

1. There were no false node‐positive patients and three possible node‐positive patients, including patients with LNM (pN+ stage), true pN0, and false pN0 stages who had LNM but were undiscovered.

2. For each patient, all lymph nodes have an equal probability of being invaded, therefore the probability of finding positive nodes is only related to the number of LNE. This is a biologically untenable assumption that refuses the effect of lymph node location, but most studies implied this.

3. For the T stage, the prevalence of LNM was similar, therefore the sensitivities of false‐negatives and true‐positives were equal. This is also a biologically untenable assumption, but it cannot be ignored unless there are more cases supporting subdivision with other characteristics, such as grade, size, and neoadjuvant therapy, which also affected the prevalence.

Based on the above assumptions, we built a model to calculate the probability of true pN0 diagnosis using the following algorithm:

1. The beta‐binomial model considers the potential correlation between the existence of positive and examined nodes. For each patient *i* in the pN+ stage in T stage *t*, LNM and LNE numbers are represented by *m*_*i,t*_ and *e*_*i,t*_, respectively. The probability that there are $m_{i,t}$ positive nodes in *e*_*i,t*_ LNE based on beta‐binomial distribution is

(1) $$P\left(m_{i,t},e_{i,t}\right)=\frac{\mathrm{Be}\left(\alpha +m_{i,t},\beta +e_{i,t}-m_{i,t}\right)}{\mathrm{Be}\left(\alpha ,\beta \right)}$$

where Be is the beta function with shape parameters α and β. $P\left(m_{i,t},e_{i,t}\right)$ is known in T stage *t*, hence we can estimate α and β by fitting the model, using the maximum likelihood.

2. We calculated the probability of false‐negative findings (P (FN)) for each pN+ patient, which means that no positive node ($m_{t}$ = 0) was found in $e_{t}$ LNE.

(2) $$\;P\left({\mathrm{FN}}_{e,t}\right)=P\left(m_{t}=0,e_{t}\right)=\frac{\mathrm{Be}\left(\alpha +0,\beta +e_{t}-0\right)}{\mathrm{Be}\left(\alpha ,\beta \right)}$$

We excluded patients with pN+ with zero negative nodes due to increased positive nodes after examining more nodes, and P (FN) was always equal to zero for these patients.

3. We estimated the number of false‐negative findings (${\mathrm{FN}}_{e,t}$) for each LNE number *e* in T stage *t*

(3) $${\mathrm{FN}}_{e,t}=\min\left\{\frac{P\left({\mathrm{FN}}_{e,t}\right)*{\mathrm{TP}}_{e,t}\;}{1-P\left({\mathrm{FN}}_{e,t}\right)},N_{e,t}\;\right\}$$

where ${\mathrm{TP}}_{e,t}$ is the number of patients with pN+, including those with zero negative nodes, and $N_{e,t}$ is the number of patients with pN0 for a given *e* in T stage *t*. We corrected some details in the previous algorithm: ${\mathrm{FN}}_{e,t}$ was equal to the minimum between estimation and $N_{e,t}$ because ${\mathrm{FN}}_{e,t}$ could not be greater than $N_{e,t}$.

4. We obtained the adjusted LNM rates for each T stage *t*

(4) $${\text{rate}}_{t}=\frac{\sum_{e}\left({\mathrm{FN}}_{e,t}+{\mathrm{TP}}_{e,t}\right)}{\sum_{e}\left({\mathrm{FN}}_{e,t}+{\mathrm{TN}}_{e,t}+{\mathrm{TP}}_{e,t}\right)}$$

where $\sum_{e}\left({\mathrm{FN}}_{e,t}+{\mathrm{TN}}_{e,t}\right)=\sum_{e}N_{e,t}$

5. Adequate staging was assessed for each patient with *e* examined nodes in the pN0 stage at T stage *t* by computing the NSS and the probability that a patient was diagnosed with true pN0 was

(5) $$\mathrm{NSS}=\frac{1-{\text{rate}}_{t}}{1-{\text{rate}}_{t}+{\text{rate}}_{t}*P\left({\mathrm{FN}}_{e,t}\right)}$$

### Precision

The precision of the reported estimates was assessed by creating 1000 bootstrap samples. The 95% confidence interval (CI) of the beta‐binomial model parameters and LNM rates before and after adjustment were formed using this bootstrap.

### Statistical analysis

More NSS resulted in better survival because there was a higher probability of patients being LNM‐free, based on our assumptions and models. Moreover, the follow‐up information not used in the model building was independent of NSS, therefore we verified NSS effectiveness by survival analysis using Kaplan–Meier curves and Cox regression. A restricted cubic spline (RCS) was used to determine the NSS thresholds as a continuous variable.

The characteristics with *p* value <0.05 from univariate models progressed into multivariate models. All the tests were two‐sided, with a significance of 5%. Python version 3.9.7 (https://www.python.org/) and R version 4.1.3 (https://www.r-project.org/) were used. We used the package “VGAM” to fit the beta‐binomial model and package “bbmle” to obtain the parameters in R.

## RESULTS

### Population characteristics

There were 1249 patients with ESCC from the SEER database, divided into pN0 (*N* = 795) and pN+ (*N* = 454) groups using an LNM rate of 35.5%. A total of 1404 patients with ESCC from our database were divided into pN0 (*N* = 743) and pN+ (*N* = 661) groups using an LNM rate of 47.1% (Table 1). The enrolled patients comprised 460 females and 789 males with a median age of 63 (interquartile range [IQR] 57–71) years from the SEER database, and 236 females and 1168 males with a median age of 60 (IQR 56–68) years from our database. Additionally, the median number of harvested lymph nodes was 12 (IQR 6–19) from the SEER database and 19 (IQR 12–26) from our database (Table 1). The median follow‐up times were 32 months with 721 cases due to ESCC and 178 cases due to other reasons in the SEER database, and 36.5 months with 772 cases due to ESCC and 15 cases due to other reasons in our database.

**TABLE 1:** Characteristics of the population from two databases

Characteristics	Levels	SEER database		Our database	
		pN0	pN1	pN0	pN1
Number		805	465	743	661
Sex (%)	Female	305 (37.9)	159 (34.2)	131 (17.6)	105 (15.9)
	Male	500 (62.1)	306 (65.8)	612 (82.4)	556 (84.1)
Race (%)	Black	151 (18.8)	106 (22.8)	NA	
	Other	103 (12.8)	59 (12.7)		
	White	551 (68.4)	300 (64.5)		
Age (M [IQR])		64 [57, 71]	63[56, 71]	60[56, 67]	60[55, 66]
Marital status (%)	Married	479 (59.5)	253 (54.4)	NA	
	No	326 (40.5)	212 (45.6)		
Site (%)	Lower	297 (36.9)	219 (47.1)	147 (19.8)	165 (25.0)
	Middle	329 (40.9)	162 (34.8)	534 (71.9)	448 (67.8)
	Other	125 (15.5)	61 (13.1)	0	0
	Upper	54 (6.7)	23 (4.9)	62 (8.3)	48 (7.3)
Tumor size (M [IQR]) (cm)		4.0 [2.5, 5.8]	4.1 [3.0, 6.0]	3.5[2.5, 4.9]	4.0[3.0 5.0]
Grade (%)	Grade I	84 (10.4)	13 (2.8)	27 (3.6)	16 (2.4)
	Grade II	425 (52.8)	220 (47.3)	559 (75.2)	491 (74.3)
	Grade III	296 (36.8)	232 (49.9)	157 (21.1)	154 (23.3)
T stage (%)	T1	171 (21.2)	39 (8.4)	116 (15.6)	34 (5.1)
	T2	169 (21.0)	69 (14.8)	171 (23.0)	117 (17.7)
	T3	340 (42.2)	211 (45.4)	260 (35.0)	255 (38.6)
	T4	125 (15.5)	146 (31.4)	196 (26.4)	255 (38.6)
Nodes_positive (M [IQR])		0	2 [1, 3]	0	2.0 [1, 4]
Chemotherapy (%)	No	345 (42.9)	208 (44.7)	414 (55.7)	283 (42.8)
	Yes	460 (57.1)	257 (55.3)	329 (44.3)	378 (57.2)
Radiation (%)	No	333 (41.4)	210 (45.2)	651 (87.6)	564 (85.3)
	Yes	472 (58.6)	255 (54.8)	92 (12.4)	97 (14.7)
Neoadjuvant chemotherapy (%)	No	502 (62.4)	352 (75.7)	687 (92.5)	603 (91.2)
	Yes	303 (37.6)	113 (24.3)	56 (7.5)	58 (8.8)
Neoadjuvant radiotherapy (%)	No	405 (50.3)	306 (65.8)	718 (96.6)	646 (97.7)
	Yes	400 (49.7)	159 (34.2)	25 (3.4)	15 (2.3)
Nodes examined (M [IQR])		11 [6, 18]	13 [7, 20]	17 [11, 24]	20[13, 27]

*Abbreviation*: M [IQR], median [interquartile range].

### Parameters of models and LNM rates of patients

The beta‐binomial parameters and adjusted LNM rates were estimated using Equation (1). The results of the parameters calculated using Equation (1) are presented in Supporting Information Table S1. The LNM rates from both databases are listed in Table 2. Subsequently, the LNM rates were adjusted using bootstrapping. The numbers of LNE in each T stage are listed in Table 2. LNM rates consistently increased with an increase in T staging after adjustment using Formula (4). The LNM rates from the SEER database were significantly lower than those from our database for each T stage (*p* < 0.001). They also followed the same trend after adjustment, except for the T4 stage, with the highest increase for patients from the SEER database (*p* < 0.001). This may be due to the inappropriate LNE numbers (M [IQR], 11 [6, 18]) for patients at stage T4 from the SEER database, in which the total number of LNE was far from adequate for standard lymphadenectomy. Compared with the LNM rates from the existing studies, both rates in the two databases increased at each T stage after adjustment, and the adjustments were significantly higher in the SEER database. This indicated that there were larger proportions of false pN0 patients in the SEER database caused by insufficient harvested LN (*p* < 0.001) (Table 2). However, for the patients in the T1 stage, the LNM rates did not significantly increase after adjustment (Table 2) in both databases, resulting from a lower prevalence of LNM in the stage T1 group.

**TABLE 2:** LNM rates and LNE of patients in different T stage from two databases

		SEER database	Our database	*p* value*
LNM rate (95% CI)	T1	0.186 (0.144–0.227)	0.228 (0.172–0.282)	<0.001
	T2	0.289 (0.244–0.338)	0.408 (0.363–0.453)	<0.001
	T3	0.383 (0.352–0.417)	0.496 (0.460–0.531)	<0.001
	T4	0.538 (0.486–0.587)	0.565 (0.526–0.601)	<0.001
Adjusted LNM rate (95% CI)	T1	0.243 (0.180–0.300)	0.269 (0.207–0.342)	<0.001
	T2	0.402 (0.336–0.470)	0.500 (0.444–0.555)	<0.001
	T3	0.512 (0.465–0.564)	0.611 (0.567–0.654)	<0.001
	T4	0.706 (0.637–0.760)	0.676 (0.631–0.722)	<0.001
Increasing rate (95% CI)	T1	0.054 (0.032–0.083)	0.041 (0.023–0.066)	<0.001
	T2	0.113 (0.078–0.152)	0.091 (0.071–0.113)	<0.001
	T3	0.128 (0.107–0.152)	0.115 (0.098–0.133)	<0.001
	T4	0.166 (0.136–0.196)	0.112 (0.095–0.130)	<0.001
Nodes examined (M [IQR])	T1	11 [6, 19]	21 [14, 26]	<0.001
	T2	11 [5, 18]	17 [11, 24]	<0.001
	T3	13 [7, 19]	18 [12, 26]	<0.001
	T4	11 [6, 18]	20 [13, 28]	<0.001

*Abbreviations*: LNE, lymph nodes examined; LNM, lymph node metastasis; M [IQR], median [interquartile range].

^a^ *t*‐test for LNM rate, adjust LNM rate and difference value of rate; Wilcox test for nodes examined.

### Probability of false‐negative findings (P (FN)) and NSS

**FIGURE 1:**
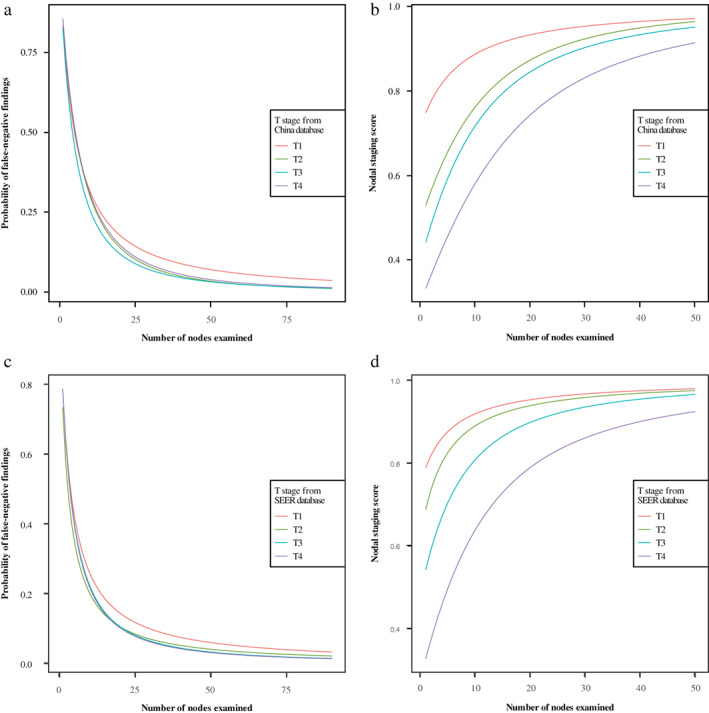
(A) The probability of false‐negative findings in pN1 patients in different T stages from the SEER database. (B) NSS according to number of nodes examined in different T stages from the SEER database. (C) The probability of false‐negative findings in pN1 patients in different T stages from China database. (D) NSS according to number of nodes examined in different T stages from China database. NSS, nodal staging score.

The probability of false‐negative nodes estimated using formula (2) was plotted (Figure 1A,C). As expected, P (FN) decreased as the LNE number increased. The probability was similar for the same number of LNE from different T stages, where the curves from the two databases followed the same pattern. The NSS as a function of nodes examined using Equation (5) was plotted (Figure 1B,D). As expected, NSS increased as the number of LNE increased. The NSS differed in the same number of LNE at different T stages: a higher T stage and a lower NSS. The NSS was the highest in stage T1 and lowest in T4, sharing the same tendency from both databases. For instance, if only one node was examined in patients with stage T1, there was a 79.1% probability of a true pN0 stage from the SEER database and a 75.0% probability from our database. However, for patients at stage T4, the NSS was 33.5% from the SEER database and 33.3% from our database (Figure 1B,D). All the P (FN) and NSS results are provided in Supporting Information Table S2.

### Verification for NSS by survival analysis

**FIGURE 2:**
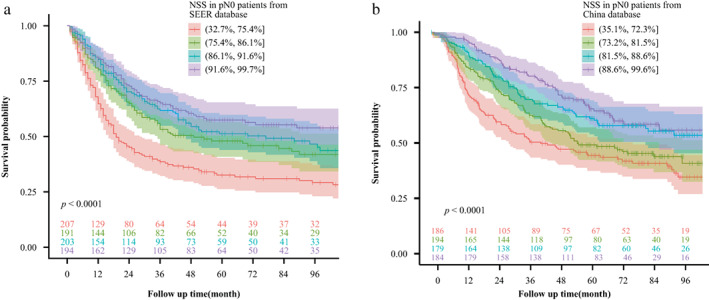
The survival of pN0 patients from two databases grouped by the quartile range of NSS: (A) SEER database and (B) China database. NSS, nodal staging score.

**FIGURE 3:**
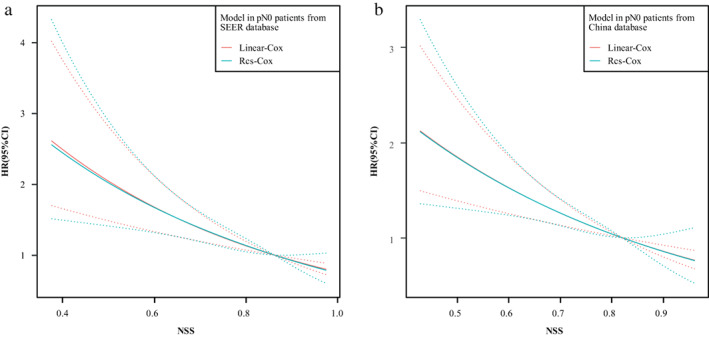
HR and NSS from linear Cox and RCS Cox with three knots. (A) Cox models that adjusted this characteristics, site, T stage, grade and chemotherapy. (B) Models from China database adjusted sex and T stage. HR, hazard ratio; NSS, nodal staging score; RCS, restricted cubic spline.

We grouped pN0 patients according to the NSS quartile range, and the Kaplan–Meier curve indicated that a higher NSS resulted in better survival (*p* < 0.001 for all T stages) (Figure 2). The 5‐year‐survival rates were 21.3%, 27.2%, 29.0%, and 33.0% from the SEER database and 36.0%, 41.2%, 45.8%, and 45.1% from our database. The Benjamini–Hochberg‐adjusted *p*‐value for pairwise comparison is presented in Supporting Information Tables S2 and S3. We included all the characteristics of patients with pN0 from the two databases in the Cox models. Age, tumor size, and LNE number were determined using their median, and NSS was a continuous variable. The results indicated that sex, tumor site, grade, T stage, chemotherapy, and NSS (hazard ratio [HR] 0.182, 95% CI 0.046–0.730, *p* = 0.016) were independent prognostic factors in patients with pN0 from the SEER database (Table 3). In addition, sex, T stage, and NSS (HR 0.215, 95% CI 0.055–0.842, *p* = 0.027) were independent prognostic factors in our database (Table 4). Multivariate RCS regression analysis with three knots adjusted for other independent prognostic factors revealed a monotonic decrease and an almost linear relationship between the HR of survival and the NSS, indicating that the prognosis improved with the increase in NSS (Figure 3).

**TABLE 3:** Cox regression for pN0 patients from SEER database

Characteristics		Univariable	*p* value	Multivariable	*p* value
		HR (95% CI)		HR (95% CI)	
Sex	Female	Reference		Reference	
	Male	1.255 (1.022–1.540)	0.030	1.317 (1.069–1.624)	0.010
Race	Black	Reference		Reference	
	Other	0.783 (0.554–1.109)	0.168	0.800 (0.556–1.149)	0.227
	White	0.740 (0.582–0.942)	0.014	0.838 (0.652–1.077)	0.167
Age†	≤64	Reference			
	>64	1.170 (0.963–1.421)	0.115		
Marital status	Married	Reference			
	No	1.147 (0.942–1.397)	0.173		
Site	Lower	Reference		Reference	
	Middle	1.458 (1.159–1.833)	0.001	1.451 (1.143–1.842)	0.002
	Other	1.546 (1.150–2.079)	0.004	1.489 (1.103–2.011)	0.009
	Upper	1.73(1.178–2.540)	0.005	1.473 (0.993–2.187)	0.054
Tumor size†	≤4.0 cm	Reference			
	> 4.0 cm	1.178 (0.968–1.433)	0.102		
Grade	Grade I	Reference		Reference	
	Grade II	1.992 (1.334–2.973)	0.001	1.914 (1.271–2.881)	0.002
	Grade III	2.099 (1.397–3.155)	<0.001	2.107 (1.384–3.208)	0.001
T stage	T1	Reference		Reference	
	T2	1.344 (0.980–1.842)	0.066	1.569 (1.132–2.176)	0.007
	T3	1.382 (1.051–1.818)	0.021	1.609 (1.139–2.273)	0.007
	T4	2.216 (1.626–3.019)	<0.001	1.781 (1.069–2.967)	0.027
Chemotherapy	No	Reference		Reference	
	Yes	0.696 (0.573–0.846)	<0.001	0.630 (0.437–0.908)	0.013
Radiation	No	Reference			
	Yes	0.837 (0.689–1.018)	0.075		
Neoadjuvant chemotherapy	No	Reference		Reference	
	Yes	0.647 (0.520–0.805)	<0.001	0.9 (0.650–1.2450)	0.524
Neoadjuvant radiotherapy	No	Reference		Reference	
	Yes	0.719 (0.590–0.876)	0.001	0.909 (0.622–1.328)	0.622
Nodes_examined^a^	≤11	Reference		Reference	
	>11	0.709 (0.583–0.863)	0.001	0.950 (0.695–1.297)	0.745
NSS		0.103 (0.054–0.197)	<0.001	0.178 (0.045–0.711)	0.015

*Abbreviation*: NSS, nodal staging score.

^a^The cutoff points for continuous variables were determined by media.

**TABLE 4:** Cox regression for pN0 patients from our database

Characteristics		Univariable		Multivariable	
		HR (95% CI)	*p* value	HR (95% CI)	*p* value
Sex	Female	Reference		Reference	
	Male	1.428 (1.057–1.928)	0.020	1.403 (1.036–1.900)	0.029
Age^a^	≤60	Reference			
	<60	1.127 (0.909–1.397)	0.274		
Site	Lower	Reference			
	Middle	1.247 (0.936–1.662)	0.132		
	Upper	0.860 (0.534–1.386)	0.536		
Tumor size†	≤3.5 cm	Reference		Reference	
	<3.5 cm	0.757 (0.611–0.938)	0.011	0.935 (0.742–1.179)	0.571
Grade	Grade I	Reference			
	Grade II	1.094 (0.613–1.951)	0.761		
	Grade III	0.802 (0.431–1.490)	0.485		
T stage	T1	Reference		Reference	
	T2	1.689 (1.103–2.586)	0.016	1.342 (0.862–2.089)	0.193
	T3	2.484 (1.672–3.688)	<0.001	1.859 (1.195–2.892)	0.006
	T4	2.428 (1.613–3.654)	<0.001	1.623 (0.961–2.740)	0.070
Chemotherapy	No	Reference			
	Yes	0.971 (0.784–1.204)	0.789		
Radiation	No	Reference		Reference	
	Yes	1.485 (1.108–1.992)	0.008	1.243 (0.922–1.675)	0.153
Neoadjuvant chemotherapy	No	Reference			
	Yes	1.214 (0.819–1.800)	0.335		
Neoadjuvant radiotherapy	No	Reference			
	Yes	1.104 (0.605–2.013)	0.747		
Nodes examined†	≤17	Reference		Reference	
	<17	1.458 (1.175–1.808)	0.001	1.123 (0.811–1.554)	0.485
NSS		0.091 (0.042–0.196)	<0.001	0.215 (0.055–0.842)	0.027

^a^The cutoff points for continuous variables were determined by media.

### Appropriate LNE numbers for evaluating the pN0 stage

Over 90% of patients must be in the true pN0 stage (NSS > 90%) to obtain an adequate number of LNE for each T stage. Based on these results, we used 1000 bootstrap samples to assess the precision of the estimated results. The numbers of LNE were eight nodes in the T1 stage, 14 nodes in T2, 21 nodes in T3, and 40 nodes in T4 from the SEER database, and 12 nodes in the T1 stage, 25 nodes in T2, 30 nodes in T3, and 45 nodes in T4 from our database (Table 5).

**TABLE 5:** Adequate number of LNE to gain NSS > 90%

	SEER database	Our database
T1	8	11
T2	14	24
T3	21	33
T4	40	41

## DISCUSSION

The LNM rate of ESCC was 42.2–51.2%^5^, ^14^, ^17^, ^29^ according to previous studies. We proposed a model with excellent interpretability based on a beta‐binomial distribution. From our model, the adjusted LNM rates increased, implying that all the patients diagnosed with the pN0 stage may be in the pN+ stage to different extents, especially in stages T2–T4 from the SEER and our database. This conclusion indicated that positive lymph nodes were associated with a sufficient number of harvested lymph nodes. As the number of LNE increased, they were more likely to experience LNM, as previous studies assumed,^14^ Our study provides powerful evidence supporting this hypothesis. We also predicted the number of LNE to avoid false‐negative results.

Previous studies have indicated that T stage, histopathology, differentiation grade, tumor size, and neoadjuvant therapy are the main factors affecting LNM in EC.^7^, ^14^, ^15^, ^16^, ^17^, ^18^ T stage was the most crucial factor for LNM in ESCC^19^: the deeper the tumor invasion, the easier it is for the tumor cells to enter the lymphatic vessels. Consequently, LNM rates have significantly improved.^20^ We therefore calculated the NSS according to the T stage by imitating Risk's study model.^5^, ^21^

LNM is an essential prognostic factor for ESCC recurrence and survival.^22^ ESCC spreads to the lymph nodes owing to the rich lymphatic network of the esophagus. Moreover, longitudinal lymphatic drainage results in wide lymph node station metastasis near the primary tumor.^19^ To our knowledge, three lymph node staging systems have been proposed as indicators of patient survival postoperatively: the AJCC nodal staging system based on numbers of positive nodes,^23^ the lymph node ratio^24^, ^25^, ^26^ (LNR, positive nodes/all nodes), and log odds of positive nodes^26^, ^27^, ^28^, ^29^ (LODDS, log [(positive nodes+0.5)/(all nodes+0.5)]), all of which are powerful indicators for the prognosis of patients with ESCC. Regardless of the number of nodes examined, the first two staging systems shared the same disadvantages (false‐negative lymph node findings). The LODDS has some interpretability for patients with pN0, but it could not stratify patients with pN0 with a significant difference.^26^, ^29^ This is because it was only a simple mathematical transformation from the total number of nodes examined.

LNE number is an essential parameter for nodal staging. For accurate staging, sufficient lymph nodes must be examined.^30^ However, the threshold of nodes that should be removed in patients with ESCC is still controversial. As the impact of the number of LNM on ESCC prognosis is gradually recognized, the minimum number of LNE for radical lymph node dissection increases gradually. The minimum number of LNE was dependent on survival analysis in several studies.^5^, ^6^, ^7^, ^20^, ^21^, ^31^, ^32^ Most studies estimated the minimum number of LNE using Kaplan–Meier curves and Cox models,^7^, ^20^, ^31^, ^32^ whereas other studies used machine learning.^5^, ^6^, ^21^ Two studies recommended the resection of at least 18 lymph nodes for the accurate staging of operable EC.^31^, ^32^ Another study recommended 20–60 nodes.^21^ Estimating the adequate number of LNE using machine learning will neglect the clinical interpretation, resulting in the loss of confidence in understanding predictive numbers. Compared to the previous nodal staging systems and LNE threshold, the major advantage of NSS is its responsiveness to patients with pN0 by quantifying the accuracy of a true pN0 diagnosis.^33^ For patients with pN0 ESCC, NSS may be the optimal selection for preoperative decision‐making, postoperative node status evaluation, and prognostic prediction.

The results indicated that NSS had no apparent thresholds for survival according to the RCS–Cox model. The number of LNE consisted of each pT stage using the NSS following the protocol by Rizk et al.^5^ More lymph nodes need to be dissected for late T stages, favoring long‐term survival, therefore we recommend the required number of nodes based on a certain desired level of NSS, such as>90%, as many previous studies did,^8^, ^9^, ^11^ using the tabular tool to calculate NSS.

The NSS is a valuable tool, but our study had several limitations. First, some information was unavailable in the SEER database, such as the surgical approach, margin status, vessel invasion, and others, which are strongly correlated with LNM and survival. Second, there were insufficient cases supporting the subdivision by grade, size, site, neoadjuvant therapy, and others, despite 1249 and 1404 patients. Further prospective clinical trials based on multicenter databases with large populations and longer follow‐ups are required to validate our findings.

## CONCLUSIONS

NSS effectively judges the accurate diagnosis of patients with pN0 ESCC, indicates survival, and determines the extent of lymphadenectomy. The number of LNE required to achieve an NSS >90% for each T stage was proposed based on two databases.

## AUTHOR CONTRIBUTIONS

Conception and design: Haitong Wang. Administrative support: PengTang and Zhentao Yu. Provision of study materials or patients: Lei Gong, Hongdian Zhang, Mingquan Ma, Peng Ren, Yufeng Qiao, Xiangming Liu, and Peng Tang. Collection and assembly of data: Haitong Wang, Yueyang Yang, Kai zhu, Ningning Zhu, and Hongdian Zhang. Data analysis and interpretation: Haitong Wang and Lei Gong. Manuscript writing and final approval of manuscript: all authors.

## CONFLICT OF INTEREST

The authors have stated no conflicts of interest regarding this manuscript.

## Supporting information


**Supporting Information Figure S1** Data‐cleaning process
**Supporting Information Table S1** Parameters for models in different T stages from two databases
**Supporting Information Table S2** The P(FN) and NSS according to nodes examined
**Supporting Information Table S3** The Benjamini–Hochberg adjusted *p* value in the log‐rank test for pairwise comparison from the SEER database
**Supporting Information Table S4** The Benjamini–Hochberg adjusted *p* value in the log‐rank test for pairwise comparison from our database
