# Supplementary material for: Development and validation of nodal staging score in pN0 patients with esophageal squamous cell carcinoma: A population study from the SEER database and a single‐institution cohort
Source: Thorac Cancer. 2022 Oct 11;13(23):3257–67. doi: 10.1111/1759-7714.14670 (PMC9715890; doi:10.1111/1759-7714.14670)
Supplement: Supplementary file 1 — Supporting Information Figure S1 Data‐cleaning process Supporting Information Table S1 Parameters for models in different T stages from two databases Supporting Information Table S2 The P(FN) and NSS according to nodes examined Supporting Information Table S3 The Benjamini–Hochberg adjusted p value in the log‐rank test for pairwise comparison from the SEER database Supporting Information Table S4 The Benjamini–Hochberg adjusted p value in the log‐rank test for pairwise comparison from our database [file TCA-13-3257-s001.docx]

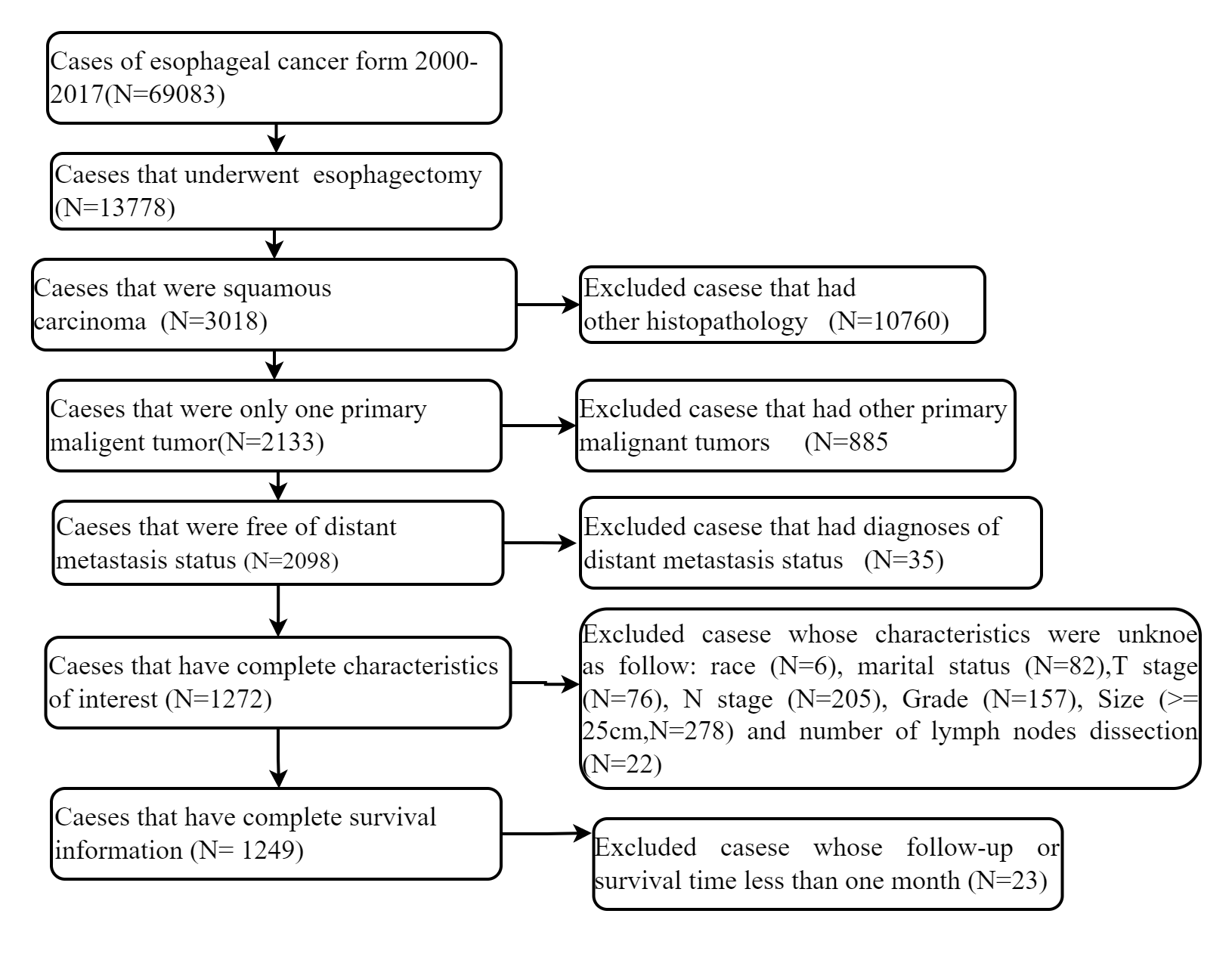
Figure S1 Data cleaning process

NOTE:

1.Site recode ICD-O-3/WHO 2008=”Esophagus”

2.Histologic Type ICD-O-3=”807x,8084,8083,8094,8052” x means any number.

3.For survival time =0 months in patients after data cleaning, we translate into 1 months

4.Python was used to clean data, and code was available upon request by contacting corresponding author.

Table S1 Parameters for models in different T stage from two databases

|  | SEER database | |  | Our database | |
| --- | --- | --- | --- | --- | --- |
|  | α (95%CI) | β (95%CI) |  | α (95%CI) | β (95%CI) |
| T1 | 1.480(0.960-3.134) | 5.281(2.732-15.499) |  | 2.023(1.186-6.630) | 11.903(5.212-54.796) |
| T2 | 1.435(1.025-2.338) | 5.216(2.936-10.665) |  | 1.979(1.495-3.077) | 10.849(7.160-19.633) |
| T3 | 1.409(1.156-1.821) | 5.046(3.551-7.341) |  | 1.895(1.530-2.427) | 10.211(7.295-14.354) |
| T4 | 1.425(1.094-1.918) | 4.907(3.259-7.728) |  | 1.831(1.494-2.340) | 9.782(7.232-13.968) |

Table S2 The P(FN) and NSS according to nodes examined

|  | SEER database | | | | | | | | Our database | | | | | | | |
| --- | --- | --- | --- | --- | --- | --- | --- | --- | --- | --- | --- | --- | --- | --- | --- | --- |
|  | T1 stage | | T2 stage | | T3 stage | | T4 stage | | T1 stage | | T2 stage | | T3 stage | | T4 stage | |
| nodes_examined | p(FN) | NSS | p(FN) | NSS | p(FN) | NSS | p(FN) | NSS | p(FN) | NSS | p(FN) | NSS | p(FN) | NSS | p(FN) | NSS |
| 1 | 0.785 | 0.789 | 0.735 | 0.688 | 0.788 | 0.542 | 0.785 | 0.327 | 0.835 | 0.750 | 0.856 | 0.530 | 0.825 | 0.442 | 0.857 | 0.333 |
| 2 | 0.644 | 0.820 | 0.576 | 0.737 | 0.641 | 0.593 | 0.636 | 0.375 | 0.714 | 0.778 | 0.740 | 0.566 | 0.693 | 0.486 | 0.742 | 0.366 |
| 3 | 0.545 | 0.843 | 0.472 | 0.774 | 0.535 | 0.636 | 0.529 | 0.419 | 0.622 | 0.801 | 0.646 | 0.599 | 0.592 | 0.525 | 0.649 | 0.397 |
| 4 | 0.472 | 0.861 | 0.398 | 0.803 | 0.455 | 0.672 | 0.449 | 0.459 | 0.549 | 0.820 | 0.568 | 0.629 | 0.511 | 0.561 | 0.572 | 0.428 |
| 5 | 0.415 | 0.876 | 0.343 | 0.825 | 0.393 | 0.704 | 0.387 | 0.496 | 0.491 | 0.836 | 0.504 | 0.657 | 0.447 | 0.594 | 0.509 | 0.457 |
| 6 | 0.371 | 0.888 | 0.301 | 0.843 | 0.344 | 0.731 | 0.338 | 0.530 | 0.443 | 0.849 | 0.449 | 0.682 | 0.394 | 0.624 | 0.455 | 0.485 |
| 7 | 0.334 | 0.898 | 0.268 | 0.858 | 0.304 | 0.754 | 0.298 | 0.561 | 0.403 | 0.861 | 0.403 | 0.705 | 0.351 | 0.651 | 0.409 | 0.511 |
| 8 | 0.304 | 0.906 | 0.241 | 0.870 | 0.271 | 0.775 | 0.266 | 0.589 | 0.369 | 0.871 | 0.364 | 0.726 | 0.314 | 0.676 | 0.370 | 0.536 |
| 9 | 0.279 | 0.913 | 0.218 | 0.881 | 0.244 | 0.792 | 0.239 | 0.615 | 0.340 | 0.880 | 0.329 | 0.745 | 0.283 | 0.698 | 0.337 | 0.560 |
| 10 | 0.258 | 0.919 | 0.200 | 0.890 | 0.221 | 0.808 | 0.216 | 0.638 | 0.315 | 0.888 | 0.300 | 0.763 | 0.257 | 0.718 | 0.307 | 0.582 |
| 11 | 0.239 | 0.925 | 0.184 | 0.898 | 0.202 | 0.822 | 0.197 | 0.660 | 0.293 | 0.895 | 0.274 | 0.779 | 0.234 | 0.737 | 0.282 | 0.603 |
| 12 | 0.223 | 0.929 | 0.170 | 0.905 | 0.185 | 0.835 | 0.180 | 0.679 | 0.274 | 0.901 | 0.251 | 0.793 | 0.214 | 0.754 | 0.259 | 0.623 |
| 13 | 0.209 | 0.934 | 0.158 | 0.911 | 0.170 | 0.846 | 0.165 | 0.697 | 0.257 | 0.907 | 0.231 | 0.807 | 0.197 | 0.769 | 0.239 | 0.641 |
| 14 | 0.196 | 0.937 | 0.148 | 0.916 | 0.157 | 0.856 | 0.153 | 0.714 | 0.242 | 0.912 | 0.213 | 0.819 | 0.181 | 0.783 | 0.221 | 0.659 |
| 15 | 0.185 | 0.941 | 0.139 | 0.921 | 0.146 | 0.865 | 0.142 | 0.729 | 0.228 | 0.916 | 0.197 | 0.830 | 0.168 | 0.796 | 0.206 | 0.675 |
| 16 | 0.175 | 0.944 | 0.130 | 0.925 | 0.136 | 0.873 | 0.132 | 0.743 | 0.216 | 0.920 | 0.183 | 0.840 | 0.156 | 0.807 | 0.191 | 0.691 |
| 17 | 0.166 | 0.946 | 0.123 | 0.929 | 0.127 | 0.880 | 0.123 | 0.756 | 0.205 | 0.924 | 0.170 | 0.850 | 0.145 | 0.818 | 0.179 | 0.705 |
| 18 | 0.158 | 0.949 | 0.116 | 0.933 | 0.119 | 0.887 | 0.115 | 0.768 | 0.195 | 0.928 | 0.159 | 0.859 | 0.136 | 0.828 | 0.167 | 0.719 |
| 19 | 0.151 | 0.951 | 0.110 | 0.936 | 0.112 | 0.893 | 0.108 | 0.779 | 0.186 | 0.931 | 0.148 | 0.867 | 0.127 | 0.837 | 0.157 | 0.732 |
| 20 | 0.144 | 0.953 | 0.105 | 0.939 | 0.105 | 0.898 | 0.102 | 0.789 | 0.177 | 0.934 | 0.139 | 0.874 | 0.119 | 0.846 | 0.147 | 0.744 |
| 21 | 0.138 | 0.955 | 0.100 | 0.942 | 0.100 | 0.904 | 0.096 | 0.799 | 0.169 | 0.937 | 0.130 | 0.881 | 0.112 | 0.854 | 0.139 | 0.755 |
| 22 | 0.132 | 0.957 | 0.096 | 0.944 | 0.094 | 0.908 | 0.091 | 0.808 | 0.162 | 0.939 | 0.123 | 0.887 | 0.106 | 0.861 | 0.131 | 0.766 |
| 23 | 0.127 | 0.959 | 0.091 | 0.947 | 0.089 | 0.913 | 0.086 | 0.816 | 0.156 | 0.941 | 0.115 | 0.893 | 0.100 | 0.868 | 0.123 | 0.776 |
| 24 | 0.122 | 0.960 | 0.088 | 0.949 | 0.085 | 0.917 | 0.082 | 0.824 | 0.149 | 0.944 | 0.109 | 0.899 | 0.094 | 0.874 | 0.117 | 0.786 |
| 25 | 0.117 | 0.962 | 0.084 | 0.951 | 0.081 | 0.920 | 0.078 | 0.831 | 0.144 | 0.946 | 0.103 | 0.904 | 0.089 | 0.880 | 0.111 | 0.795 |
| 26 | 0.113 | 0.963 | 0.081 | 0.952 | 0.077 | 0.924 | 0.074 | 0.838 | 0.138 | 0.948 | 0.097 | 0.908 | 0.085 | 0.885 | 0.105 | 0.803 |
| 27 | 0.109 | 0.964 | 0.078 | 0.954 | 0.073 | 0.927 | 0.070 | 0.844 | 0.133 | 0.949 | 0.092 | 0.913 | 0.081 | 0.890 | 0.100 | 0.811 |
| 28 | 0.105 | 0.965 | 0.075 | 0.956 | 0.070 | 0.930 | 0.067 | 0.850 | 0.129 | 0.951 | 0.087 | 0.917 | 0.077 | 0.895 | 0.095 | 0.819 |
| 29 | 0.102 | 0.966 | 0.072 | 0.957 | 0.067 | 0.933 | 0.064 | 0.856 | 0.124 | 0.953 | 0.083 | 0.921 | 0.073 | 0.900 | 0.090 | 0.826 |
| 30 | 0.099 | 0.967 | 0.070 | 0.959 | 0.064 | 0.936 | 0.062 | 0.861 | 0.120 | 0.954 | 0.079 | 0.924 | 0.070 | 0.904 | 0.086 | 0.833 |
| 31 | 0.096 | 0.968 | 0.067 | 0.960 | 0.062 | 0.938 | 0.059 | 0.866 | 0.116 | 0.955 | 0.075 | 0.928 | 0.067 | 0.908 | 0.082 | 0.839 |
| 32 | 0.093 | 0.969 | 0.065 | 0.961 | 0.059 | 0.940 | 0.057 | 0.871 | 0.113 | 0.957 | 0.072 | 0.931 | 0.064 | 0.911 | 0.079 | 0.845 |
| 33 | 0.090 | 0.970 | 0.063 | 0.962 | 0.057 | 0.943 | 0.054 | 0.875 | 0.109 | 0.958 | 0.068 | 0.934 | 0.061 | 0.915 | 0.075 | 0.851 |
| 34 | 0.088 | 0.971 | 0.061 | 0.963 | 0.055 | 0.945 | 0.052 | 0.879 | 0.106 | 0.959 | 0.065 | 0.937 | 0.058 | 0.918 | 0.072 | 0.856 |
| 35 | 0.085 | 0.972 | 0.059 | 0.965 | 0.053 | 0.947 | 0.050 | 0.883 | 0.103 | 0.960 | 0.062 | 0.939 | 0.056 | 0.921 | 0.069 | 0.861 |
| 36 | 0.083 | 0.973 | 0.058 | 0.966 | 0.051 | 0.948 | 0.049 | 0.887 | 0.100 | 0.961 | 0.060 | 0.942 | 0.054 | 0.924 | 0.066 | 0.866 |
| 37 | 0.081 | 0.973 | 0.056 | 0.966 | 0.049 | 0.950 | 0.047 | 0.891 | 0.097 | 0.962 | 0.057 | 0.944 | 0.052 | 0.927 | 0.063 | 0.871 |
| 38 | 0.079 | 0.974 | 0.055 | 0.967 | 0.047 | 0.952 | 0.045 | 0.894 | 0.095 | 0.963 | 0.055 | 0.946 | 0.050 | 0.929 | 0.061 | 0.875 |
| 39 | 0.077 | 0.975 | 0.053 | 0.968 | 0.046 | 0.953 | 0.044 | 0.897 | 0.092 | 0.964 | 0.053 | 0.948 | 0.048 | 0.932 | 0.059 | 0.879 |
| 40 | 0.075 | 0.975 | 0.052 | 0.969 | 0.044 | 0.955 | 0.042 | 0.900 | 0.090 | 0.965 | 0.050 | 0.950 | 0.046 | 0.934 | 0.056 | 0.883 |
| 41 | 0.073 | 0.976 | 0.050 | 0.970 | 0.043 | 0.956 | 0.041 | 0.903 | 0.088 | 0.966 | 0.048 | 0.952 | 0.044 | 0.936 | 0.054 | 0.887 |
| 42 | 0.071 | 0.976 | 0.049 | 0.971 | 0.041 | 0.957 | 0.039 | 0.906 | 0.085 | 0.967 | 0.047 | 0.954 | 0.043 | 0.938 | 0.052 | 0.891 |
| 43 | 0.070 | 0.977 | 0.048 | 0.971 | 0.040 | 0.959 | 0.038 | 0.909 | 0.083 | 0.968 | 0.045 | 0.956 | 0.041 | 0.940 | 0.050 | 0.894 |
| 44 | 0.068 | 0.977 | 0.047 | 0.972 | 0.039 | 0.960 | 0.037 | 0.911 | 0.081 | 0.968 | 0.043 | 0.957 | 0.040 | 0.942 | 0.049 | 0.898 |
| 45 | 0.067 | 0.978 | 0.046 | 0.973 | 0.038 | 0.961 | 0.036 | 0.914 | 0.079 | 0.969 | 0.042 | 0.959 | 0.039 | 0.944 | 0.047 | 0.901 |
| 46 | 0.065 | 0.978 | 0.045 | 0.973 | 0.037 | 0.962 | 0.035 | 0.916 | 0.078 | 0.970 | 0.040 | 0.960 | 0.037 | 0.946 | 0.045 | 0.904 |
| 47 | 0.064 | 0.979 | 0.044 | 0.974 | 0.036 | 0.963 | 0.034 | 0.918 | 0.076 | 0.971 | 0.039 | 0.961 | 0.036 | 0.948 | 0.044 | 0.907 |
| 48 | 0.062 | 0.979 | 0.043 | 0.974 | 0.035 | 0.964 | 0.033 | 0.921 | 0.074 | 0.971 | 0.037 | 0.963 | 0.035 | 0.949 | 0.042 | 0.910 |
| 49 | 0.061 | 0.980 | 0.042 | 0.975 | 0.034 | 0.965 | 0.032 | 0.923 | 0.073 | 0.972 | 0.036 | 0.964 | 0.034 | 0.951 | 0.041 | 0.912 |
| 50 | 0.060 | 0.980 | 0.041 | 0.975 | 0.033 | 0.966 | 0.031 | 0.925 | 0.071 | 0.972 | 0.035 | 0.965 | 0.033 | 0.952 | 0.040 | 0.915 |

Abbreviations: NSS, nodal staging score.

Table S3 The Benjamini-Hochberg adjusted *P*-value in Log-rank test for pairwise comparison from SEER database

|  | (32.7%,75.4%] | (75.4%~86.1%] | (86.1%~91.6%] |
| --- | --- | --- | --- |
| (75.4%~86.1%] | 0.001 | - | - |
| (86.1%~91.6%] | 0 | 0.359 | - |
| (91.6%~99.7%] | 0 | 0.080 | 0.338 |

Table S4 The Benjamini-Hochberg adjusted *P*-value in Log-rank test for pairwise comparison from our database

|  | (35.1%,73.2%] | (73.2%,81.5%] | (81.5%,88.6%] |
| --- | --- | --- | --- |
| (73.2%,81.5%] | 0.099 | - | - |
| (81.5%,88.6%] | 0.000 | 0.052 | - |
| (88.6%,99.6%] | 0.000 | 0.002 | 0.270 |
